# Effects of low‐dust forages on dust exposure, airway cytology, and plasma omega‐3 concentrations in Thoroughbred racehorses: A randomized clinical trial

**DOI:** 10.1111/jvim.16598

**Published:** 2022-12-07

**Authors:** Carla J. Olave, Kathleen M. Ivester, Laurent L. Couetil, John Burgess, Jae Hong Park, Abhijit Mukhopadhyay

**Affiliations:** ^1^ Department of Veterinary Clinical Sciences, College of Veterinary Medicine Purdue University West Lafayette Indiana USA; ^2^ Department of Nutrition Science, College of Health and Human Sciences Purdue University West Lafayette Indiana USA; ^3^ School of Health Sciences, College of Health and Human Sciences Purdue University West Lafayette Indiana USA

**Keywords:** airway, asthma, haylage, inflammation, neutrophils, resolution, respiratory tract

## Abstract

**Background:**

Racehorses commonly develop evidence of mild asthma in response to dust exposure. Diets deficient in omega‐3 polyunsaturated fatty acids (Ω‐3) might exacerbate this response.

**Hypothesis:**

To compare dust exposure, bronchoalveolar lavage fluid (BALF) cytology, and plasma Ω‐3 and specialized pro‐resolving mediators (SPM) concentrations amongst racehorses fed dry hay, steamed hay, and haylage.

**Animals:**

Forty‐three Thoroughbred racehorses.

**Methods:**

Prospective clinical trial. Horses were randomly assigned to be fed dry hay, steamed hay, or haylage for 6 weeks. Measures of exposure to dust in the breathing zone were obtained twice. At baseline, week‐3, and week‐6, BALF cytology was examined. Plasma lipid profiles and plasma SPM concentrations were examined at baseline and week 6. Generalized linear mixed models examined the effect of forage upon dust exposure, BALF cytology, Ω‐3, and SPM concentrations.

**Results:**

Respirable dust was significantly higher for horses fed hay (least‐square mean ± s.e.m. 0.081 ± 0.007 mg/m^3^) when compared with steamed hay (0.056 ± 0.005 mg/m^3^, *P* = .01) or haylage (0.053 ± 0.005 mg/m^3^, *P* < .01). At week 6, BALF neutrophil proportions in horses eating haylage (3.0% ± 0.6%) were significantly lower compared with baseline (5.1 ± 0.7, *P* = .04) and horses eating hay (6.3% ± 0.8%, *P* < .01). Plasma eicosapentaenoic acid to arachidonic acid ratios were higher in horses eating haylage for 6 weeks (0.51 ± 0.07) when compared with baseline (0.34 ± 0.05, *P* < .01) and horses eating steamed (0.24 ± 0.02, *P* < .01) or dry hay (0.25 ± 0.03, *P* < .01).

**Conclusions and Clinical Importance:**

Steamed hay and haylage reduce dust exposure compared with dry hay, but only haylage increased the ratio of anti‐inflammatory to pro‐inflammatory lipids while reducing BAL neutrophil proportions within 6 weeks.

**Abbreviations**
BALF: bronchoalveolar lavage fluid
DHA: docosahexaenoic acid
EPA: eicosapentaenoic acid
LAL: limulus amebocyte lysate
LOD: limit of detection
MA: mild asthma
PM: particulate matter
PM_1_
: particulate matter ≤1 μm
PM_10_
: particulate matter ≤10 μm
PM_2.5_
: particulate matter ≤2.5 μm
PM_2.5‐10_
: particulate matter from 2.5 to 10 μm
PUFAs: polyunsaturated fatty acids
RVD1: resolving D1
RVE1: resolving E1
SPM: specialized pro‐resolving mediators
Ω‐3: omega‐3
Ω‐6: omega‐6

## 1 INTRODUCTION

Exposure to organic dust is central to the development of mild asthma in horses (MA),^1^, ^2^ a commonly encountered,^1^, ^3^, ^4^, ^5^ performance‐limiting^1^, ^4^ inflammatory respiratory disease. Compared with horses at pasture, stabled horses are exposed to higher concentrations of organic dust,^2^, ^6^ which includes varying quantities of immunoreactive bacterial and fungal derived products such as endotoxin and β‐glucan depending upon the bedding and feed used.^7^ In racehorses, bronchoalveolar lavage fluid (BALF) mast cell proportions vary with respirable β‐glucan exposure, whereas BALF neutrophil proportions increase with increasing respirable dust exposure, a response modulated by concurrent endotoxin exposure.^1^

Racehorses remain in their stall an average of 23 hours/day and are mainly fed dry hay. Other forages with higher moisture content and lower dust production are available. Haylage, a conserved forage that is harvested at a higher moisture content than hay, reduces exposure of horses to respirable dust by 60% to 70% when compared with dry hay.^8^, ^9^ Alternatively, dry hay can be steamed to reduce respirable dust release in vitro by 95%,^10^ and bacterial and mold content by 99%,^10^, ^11^ Outside of the laboratory, the efficacy of low‐dust forages to reduce dust exposure and airway inflammation remains unknown.

Resolution of inflammation is often prolonged and incomplete once low‐dust management is instituted.^12^, ^13^, ^14^ Chronic inflammation present in the asthmatic airways might result not only from repetitive exposure to triggering stimuli but also from impaired pro‐resolving pathways.^13^, ^14^ Specialized pro‐resolving mediators (SPM) play an active role in the return to tissue homeostasis after an inflammatory event by reducing leukocyte recruitment, inducing neutrophil apoptosis, and enhancing efferocytosis at the site of inflammation.^15^, ^16^, ^17^, ^18^

SPM are derived from essential omega‐3 (Ω‐3), and to a lesser extent, omega‐6 (Ω‐6) polyunsaturated fatty acids (PUFAs)^19^; the relative availability of each are determined by dietary intake. Arachidonic acid and its inflammatory derivatives, prostaglandins, thromboxane, and leukotrienes, are derived from Ω‐6, and excessive Ω‐6 intake has been associated with increased inflammatory diseases in people.^20^ Increased intake of Ω‐3 might help mitigate airway inflammation in humans^21^ and horses with severe asthma.^12^ Racehorses are typically fed dry hay, which contains less α‐linolenic acid (Ω‐3) when compared with pasture and haylage.^22^ Therefore, in addition to lowering dust exposure, feeding haylage to racehorses might provide further benefit because of higher Ω‐3 content.

Consequently, we hypothesized that feeding haylage or steamed hay to racing Thoroughbreds in place of dry hay would reduce breathing‐zone measures of respirable dust, β‐glucan, and endotoxin, and would result in significantly lower BALF proportions of neutrophils and mast cells. We expected that horses fed haylage would have higher plasma concentrations of Ω‐3 and SPMs and display faster resolution of airway inflammation.

## 2 MATERIAL AND METHODS

### 2.1 Experimental design

A prospective randomized feed trial was designed to compare respirable dust exposure and airway cytology between horses fed dry hay, haylage, or steamed hay for 6 weeks. The study was performed during two racing meets between May 2018 and October 2019. Trainers allowed horses to enroll in the study by providing informed consent and completing a short questionnaire detailing the length of ownership, vaccination history, and any history of respiratory illness. Horses with signs of respiratory infection or systemic illness (fever, abnormal hematology, decreased appetite) were excluded. Upon enrollment, horses were allocated to receive dry hay, haylage, or steamed hay as their sole source of forage using simple randomization through computer‐generated random numbers. Vaccination against *Clostridium botulinum* type‐B before assignment to haylage was offered to the trainer. No other change was made to the horses' management. Horses continued to be fed the same amount of grain, mineral, and vitamin supplements according to the trainer's preference. All horses were bedded on sawdust. Horses fed hay and steamed hay were eligible for re‐enrollment.

At baseline, physical examination, blood collection, endoscopy of the respiratory tract, and bronchoalveolar lavage (BAL) were performed. For those horses assigned to receive haylage, the forage was gradually introduced whereas hay was gradually excluded from the diet over the course of 7 days. Those horses assigned to dry and steamed hay groups continued to be fed the hay they received before enrollment. Trainers were provided with haylage (C&M Forage) and with a commercially available hay steamer (Haygain) free of charge and instructed on its use. Haylage with visible mold growth was discarded, and trainers were instructed to feed haylage bales within 3 days once the plastic wrap was opened. Horses remained on the assigned forage for 6 weeks. After 3 and 6 weeks on the assigned forage, breathing zone measures of respirable dust exposure, physical and endoscopic examinations, and BAL were performed. During the study, training and racing schedules were continued as usual while adhering to recommended drug elimination times^23^ for sedation and topical lidocaine used for the BAL procedure.

#### 2.1.1 Dust exposure measurements

##### Sampling and analysis of respirable dust

Exposures to respirable dust were assessed using methods as described.^24^ Briefly, a conventional respirable sampler consisting of a respirable cyclone (P225‐01‐02, SKC, Inc., Eighty‐Four, PA) to remove dust larger than the respirable fraction and was fitted to a 3‐piece cassette (SKC, Inc., Eighty Four, PA) which housed a polyvinyl chloride filter (GLA5000, 37 mm diameter). To locate the inlet of the sampler in the breathing zone, the sampler was fixed on the noseband of the halter. The sampler was connected to a sampling pump (AirChek‐2000, SKC, Inc., Eighty Four, PA) fixed on a surcingle placed around the girth of the horse. Flexible tubing (Tygon, Saint Gobain, France) secured to the mane and forelock of the horse was used to connect the sampler to the sampling pump. The sampling flow rate and time were 2.5 L/min and 3 hours, respectively. To calculate the weight of respirable dust collected on the filter, the weights of filters before and after sampling were measured using the microbalance (AT250, Mettler Toledo, Columbus, OH). The average of three weights of the filter before sampling was subtracted from the average of three weights of the filter after sampling. The weight of respirable dust lighter than 0.02 μg was considered below the limit of detection (LOD).^25^ A time‐weighted average concentration was calculated by dividing the weight of respirable dust by the sampling volume (=sampling flow rate × sampling time). The lower LOD for respirable dust concentration was 0.047 mg/m^3^. Subsequently, polyvinyl chloride filters were stored at −20°C until elution for β‐glucan and endotoxin analyses.

##### Real‐time measurement of particulate matter

Concentrations of particulate matter (PM) in the horse's breathing zone were measured in real‐time using a PM monitor (OPC‐N2, Alphasense, Essex, UK). The PM monitor was secured to the crown piece of a break‐away halter and its inlet was connected to flexible tubing that extended to the noseband of the halter to sample PM in the breathing zone of the horse. PM concentrations were classified by their size. Specifically, mass concentrations of PM_1_ (≤1 μm), PM_2.5_ (≤2.5 μm), PM_2.5‐10_ (from 2.5 to 10 μm), and PM_10_ (≤10 μm) were obtained. During the 3‐hour measurement period, the horse was free to move around the stall, eat, and drink as usual.

#### 2.1.2 Βeta‐glucan and endotoxin analysis

The content of β‐glucan and endotoxin in respirable dust was measured using a kinetic chromogenic Limulus amebocyte lysate technique (NexGen PTS0, Charles River Laboratories, Wilmington, MA) as previously described.^1^ A time‐weighted average exposure was calculated by dividing the product of the measured concentration of β‐glucan and endotoxin and the volume of eluant by the volume of air sampled [(pg or EU/mL * mL eluant)/sampling flow rate × sampling time)]. The lower LODs of the measurements were 43.7 pg/m^3^ and 0.02 EU/m^3^ for β‐glucan and endotoxin, respectively.

### 2.2 Clinical score

Upon physical examination, a clinical score (range: 0‐21) based on cough, nasal discharge, respiratory efforts, and auscultation was determined as described.^12^

#### 2.2.1 Endoscopic examination

Horses were restrained with a nose twitch and a flexible video‐endoscope with 7.9 mm outer diameter was passed through the ventral meatus to the level of the pharynx. A score was assigned to the degree of pharyngeal lymphoid hyperplasia present from 0 (no follicles) to 4 (numerous, large, edematous follicles).^2^ Any upper respiratory tract abnormality was noted. Tracheal mucus was scored from 0 (no mucus) to 4 (large, pool‐forming).^26^ To facilitate BAL, the carina and larynx were sprayed with a 0.4% lidocaine solution as the endoscope was removed (20‐30 mL at each site).

#### 2.2.2 Bronchoalveolar lavage

Horses were sedated by intravenous injection of xylazine hydrochloride (0.2‐0.5 mg/kg; AnaSed, Akorn Animal Health, Lake Forest, IL) and butorphanol (0.02‐0.04 mg/kg; Torbugesic, Zoetis, Parsippany‐Troy Hills, NJ). A sterile BAL tube (300 cm long; 10 mm OD; Bivona Medical Technologies, Gary, IN) with an inflatable cuff was passed through the nose and wedged into a peripheral bronchus. Five 50 mL aliquots of sterile 0.9% sodium chloride solution were infused and recovered manually using 60 mL syringes. The BALF was filtered with gauze and immediately placed on ice. Samples were processed within 1 hour of collection. Cytological specimens were prepared by cytospin centrifugation and processed with modified Wright's stain. Differential cell counts were performed on 600 cells by a single observer (CO), including at least five microscopic fields^27^; epithelial cells were not included in the cells counted. Threshold values were used to determine the presence of airway inflammation in BALF were neutrophils >5%, mast cells >2%, or eosinophils >1%.^3^

#### 2.2.3 Fatty acid analysis

Venous blood was collected from the jugular vein into evacuated tubes containing EDTA for hematology at baseline and plasma separation at baseline, week‐3 and week‐6. Plasma samples were stored at −80°C until analysis. Fatty acid analysis of plasma samples was performed on freshly thawed aliquots after extraction of lipids by the Folch method,^28^ isolation of phospholipids by solid phase extraction using silica cartridges, and methylation utilizing boron trifluoride and gas chromatography.^29^ Fatty acid measured included linoleic acid, α‐linolenic acid, arachidonic acid, eicosapentaenoic acid (EPA), and docosahexaenoic acid (DHA).

#### 2.2.4 Specialized pro‐resolving mediator analysis

Plasma samples from baseline and week‐6 were stored at −80°C until analyses. Selected SPM were measured using equine‐specific ELISA kits for resolving D1 (RvD1) and resolving E1 (RvE1; MyBiosource, San Diego, CA) according to the manufacturer's instructions, using a plate reader (BioTek, Winooski, VT). Measures were performed in duplicate, and the average was recorded.

### 2.3 Data analysis

#### 2.3.1 Statistical analysis

Generalized linear mixed models^1^ were constructed to examine the effect of forage assignment upon dust exposure, BALF cytology, SPM concentrations, and proportional composition of PUFAs, controlling for age (fixed covariate), trainer (random effect), and repeated measures (random effects). Model assumptions of residual distributions were checked graphically. Distribution and link functions specified for each outcome measure are listed in Table S1. Data are displayed on the original scale. Significance of post hoc pairwise comparisons was controlled by Tukey's post hoc method, and an adjusted *P*‐value of <.05 was considered significant. Respirable dust exposure values below the LOD were assigned a value equal to the lower LOD divided by the square root of 2.^25^ Data analyses were performed using Proc GLIMMIX SAS v.9.4 (SAS Institute, Cary, NC), and graphs were made with GraphPad Prism 8 (GraphPad Software, San Diego, CA), MetaboAnalyst 3.0q (http://www.metaboanalyst.ca), and ProcSGPLOT, SAS v9.4.

#### 2.3.2 Sample size calculations

Preliminary data demonstrated a mean BALF neutrophil proportion of 4.75% (SD = 3.95%) in horses racing at the racetrack^1^ and a mean BALF neutrophil proportion of 0.75% (4‐fold reduction from baseline) in horses fed haylage for 6 weeks.^9^ A sample size of 15 horses per group, or 45 total horses, would provide a 90% power to detect a statistically significant difference between groups with *P* ≤ .05. To allow for subject dropout over the 6‐week enrollment period, a target enrollment of 20 per group for a total of 60 horses was planned.

## 3 RESULTS

Data were reported according to the recommendations from the 2010 CONSORT statement (http://www.consort-statement.org/). Raw data are reported as mean ± SD, while results of statistical models are reported as least‐square means ± SE.

### 3.1 Horses

**FIGURE 1:**
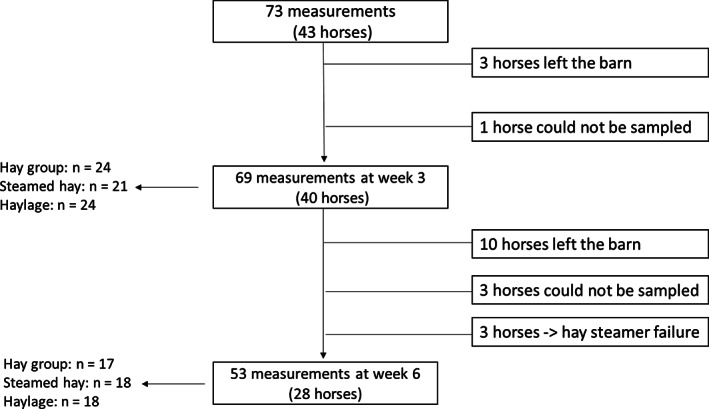
Flow diagram of horses that participated in the trial with forage assignment

Forty‐three horses were enrolled in the study. In response to inadequate enrollment, horses randomly assigned to be fed dry or steamed hay were eligible for re‐enrollment. Horses randomly assigned to be fed haylage were not eligible for re‐enrollment within the calendar year because of unknown duration of potentially altered PUFA profiles. This enrollment strategy led to re‐enrollment of 21 horses, with 12 horses re‐enrolled once, and 9 horses re‐enrolled twice. Seventy‐three measurements were performed at baseline, 69 at week‐3, and 53 at week‐6 (Figure 1, Table S2). Thirteen horses left the barn for causes not related to the study, four could not be sampled because of conflicts with the barn's schedule, and three horses did not receive the assigned forage (steamed hay) because of an error operating the hay steamer. Five stallions (12%), 20 geldings (47%), and 18 mares (42%) were enrolled, and the age was 4.0 ± 1.7 years (mean ± SD). Four trainers participated, 1 with 33 horses (77%), 1 with 6 horses (14%), and 2 with 2 horses each (4.5%). Clinical and BAL variables measured at baseline for horses enrolled in the study did not differ between forage groups (Table 1). At baseline, cytologic evidence of MA was observed in 66 out of 73 measurements (90%). Mast cell inflammation was most commonly observed (36%), followed by mixed inflammation (33%), neutrophilic inflammation (18%), and eosinophilic inflammation (4%).

**TABLE 1:** Clinical and airway cytology variables of horses assigned to each forage group at baseline (data presented as mean ± SD)

	Hay	Steamed Hay	Haylage	*P*‐value
*Clinical variables*				
Number of horses	28	22	23	
Age	3.6 ± 1.2	3.9 ± 1.2	4.2 ± 2.0	.36
Sex	16 geldings, 8 mares, 4 stallions	12 geldings, 10 mares	7 geldings, 15 mares, 1 stallion	
Respiratory rate (breaths per min)	20 ± 2	21 ± 5	19 ± 4	.29
Clinical score	2 ± 1	2 ± 1	2 ± 1	.54
Mucus score	1 ± 1	1 ± 1	1 ± 1	.77
*Bronchoalveolar lavage fluid cytology*				
Total nucleated cell count (cells/μL)	303 ± 112	291 ± 64	273 ± 104	.49
Macrophages (%)	50.3 ± 9.7	49.1 ± 9.6	50.0 ± 9.0	.88
Lymphocytes (%)	40.0 ± 8.2	41.0 ± 7.0	42.1 ± 8.7	.65
Neutrophils (%)	4.8 ± 2.9	5.2 ± 4.0	5.4 ± 3.6	.84
Mast cells (%)	2.9 ± 1.5	2.9 ± 1.3	2.5 ± 1.0	.86
Eosinophils (%)	2.6 ± 8.1	2.1 ± 7.7	1.0 ± 1.0	.57

### 3.2 Effect of forage on airway cytology

**FIGURE 2:**
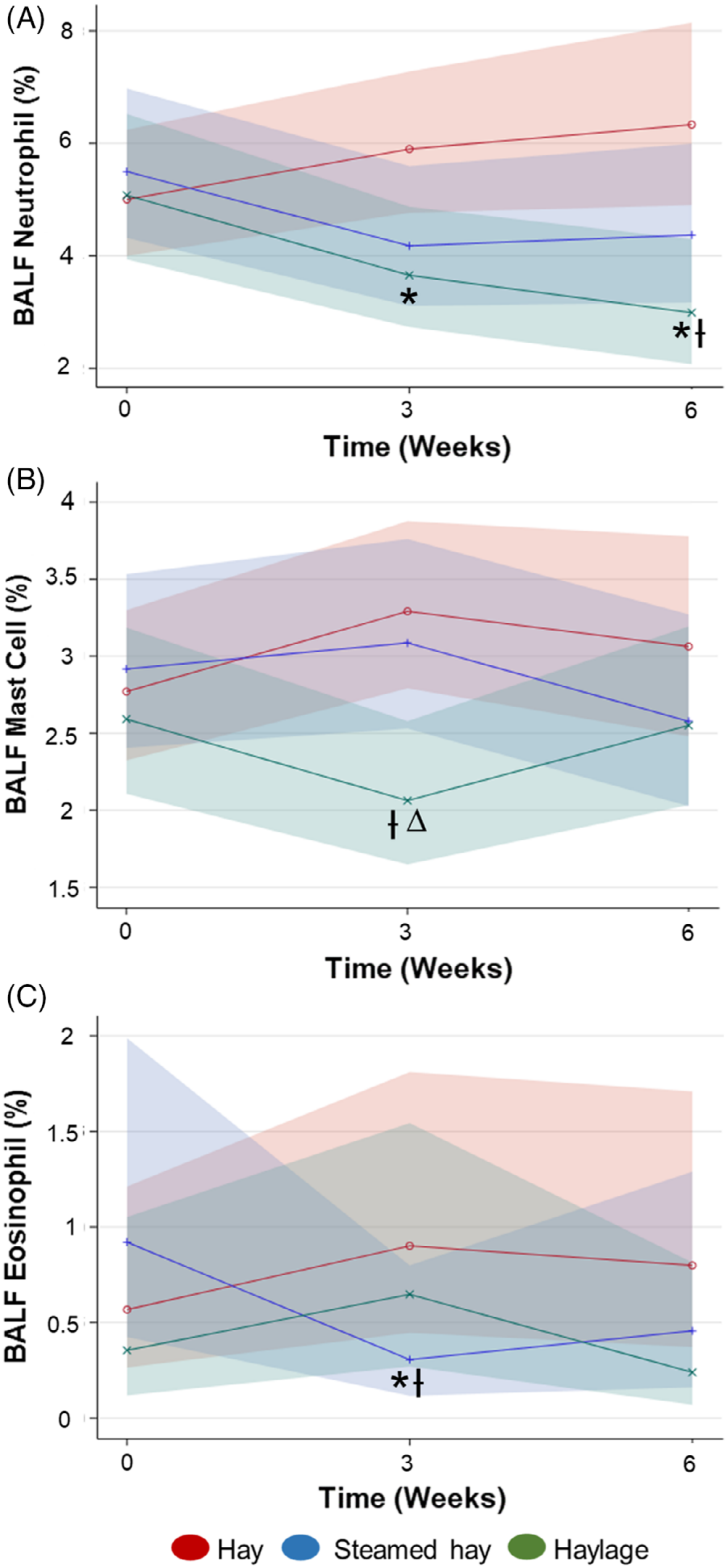
BALF cytology by forage over time. (A) Generalized linear repeated‐measures model of BALF neutrophil proportions (%) by forage group over time. *Different from baseline (*P* = .04). Ɨ Different from hay at week 6 (*P* = .02). (B) Generalized linear mixed model of BALF mast cell proportions (%) by forage group over time. Ɨ Different from hay at week‐3 (*P* = .003). ∆ Different from steamed hay at week‐3 (*P* = .02). (C) Generalized linear mixed model of BALF eosinophil proportions (%) by forage group over time. *Different from baseline (*P* = .03). Ɨ Different from hay at week‐3 (*P* = .04). Solid line = mean response, and band = 95% confidence interval. Means, confidence intervals, and *P*‐values calculated from generalized linear repeated‐measures regression models assuming binomial distribution under logit link function and controlling for age and trainer

**FIGURE 3:**
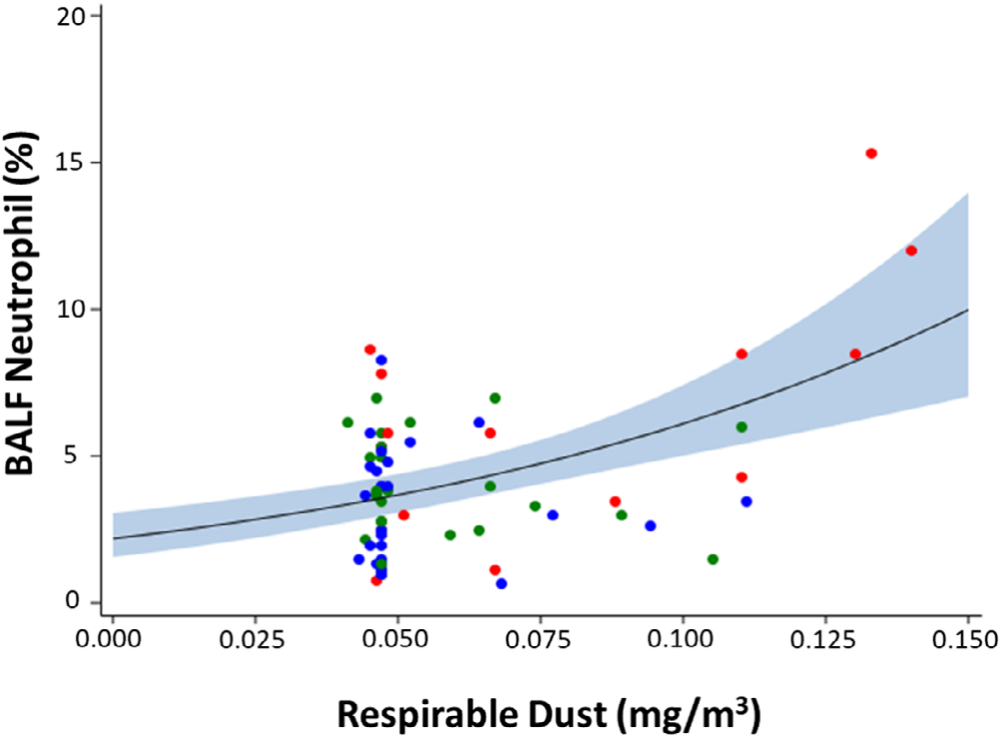
Association between BALF neutrophil proportions (%) and breathing zone respirable dust exposure. Generalized linear repeated‐measures model of BALF neutrophil proportion versus respirable dust exposure. Solid line = predicted mean response fit at age = 4.47 years. Band = 95% confidence interval of the mean response. Red dots = hay; blue dots = steamed hay; green dots = haylage. Means, confidence intervals, and *P*‐values calculated from generalized linear repeated‐measures regression models assuming binomial distribution under logit link function and controlling for age and trainer

Horses eating haylage had lower BALF neutrophil proportions at week‐3 (*P* = .02) and week‐6 (*P* < .01) compared with horses eating hay (Figure 2A). After 6 weeks, horses fed haylage had lower proportions of BALF neutrophils compared with baseline (*P* = .04). Horses consuming steamed hay had no decrease in BALF neutrophil proportions after 3 and 6 weeks compared with baseline (*P* = .20 and *P* = .46, respectively). Neutrophil proportions in BALF were positively associated with increasing respirable dust exposure (*P* < .001; Figure 3).

Horses eating haylage exhibited significantly lower BALF mast cell proportions at week‐3 when compared with horses eating hay (*P* = .003) and steamed hay (*P* = .02), however, mast cells returned to baseline levels by week‐6 (Figure 2B). No significant change in BALF mast cell proportion was detected in horses eating hay or steamed hay.

Horses eating steamed hay exhibited a decrease in BALF eosinophil proportions after 3 weeks compared with baseline (*P* = .03) and horses eating hay (*P* = .04; Figure 2C). However, by week‐6 BALF eosinophil proportions in horses fed steamed hay were not significantly different from horses fed hay. No significant change in BALF eosinophil proportions was detected in horses eating dry or haylage.

### 3.3 Effect of forage on dust exposure

**FIGURE 4:**
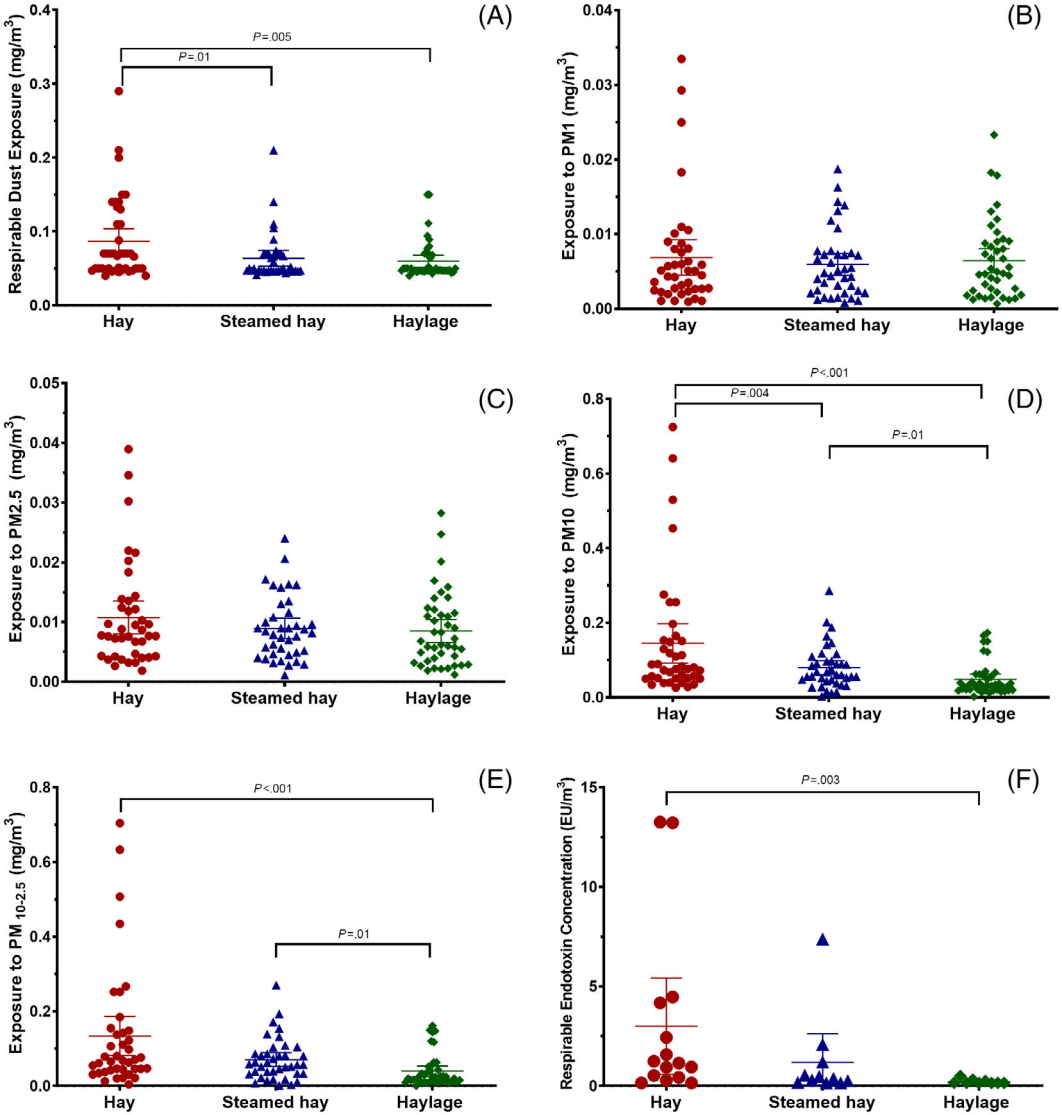
Scatter plot of breathing zone respirable dust exposure, particle matter (PM) concentrations, and respirable endotoxin exposure in horses fed dry hay, steamed hay, or haylage. (A) Respirable dust exposure. (B) Particulate matter with a diameter ≤1 μm (PM_1_) concentration. (C) PM_2.5_ concentration. (D) PM_10_ concentration. (E) PM_2.5‐10_ concentration. (F) Respirable endotoxin exposure. Horizontal bars indicate mean and 95% confidence interval. Means, confidence intervals, and *P*‐values calculated from generalized linear repeated‐measures regression models assuming lognormal distribution and controlling for age and trainer

Respirable dust concentrations measured during the second year of sampling were not included in the analyses because of a technical error resulting in weights being recorded to the nearest 0.1 mg rather than 0.01 mg, effectively rendering all measures below the lower LOD. During the first year, 72 measurements of respirable dust were performed, 48 of those measurements were under the lower LOD, 8 in the horses fed hay, 19 in the horses fed steamed hay, and 21 in the horses eating haylage. Respirable dust exposure was significantly higher when dry hay (n = 17) was fed compared with when steamed hay (n = 28; *P* = .01) or haylage (n = 27; *P* = .005) was fed (Figure 4A).

Mean exposure to PM_1_ and PM_2.5_ did not differ with forage assignment (*P* = .92 and *P* = .28, respectively; Figure 4B,C). Mean PM_10_ and PM_2.5‐10_ exposures were significantly higher in horses eating hay (0.13 ± 0.16 mg/m^3^, *P* < .001 and 0.12 ± 0.15 mg/m^3^, *P* < .001) and steamed hay (0.08 ± 0.06 mg/m^3^, *P* = .01 and 0.07 ± 0.06 mg/m^3^, *P* = .01) when compared with horses eating haylage (0.05 ± 0.05 and 0.04 ± 0.05 mg/m^3^, Figure 4D,E). PM_10_ exposure was significantly higher for horses eating hay when compared with horses eating steamed hay (*P* = .004; Figure 4D).

### 3.4 Effect of forage on respirable β‐glucan and endotoxin exposures

Fifty‐one respirable dust filter samples from week‐6 were available for β‐glucan analysis. Forty‐five samples were under the lower LOD of the β‐glucan assay (43.7 pg/m^3^). Respirable β‐glucan exposure was not different between groups (Hay = 48.3 ± 4.2 pg/m^3^; steamed hay = 46.4 ± 6.3 pg/m^3^; haylage = 44.5 ± 2.2 pg/m^3^; *P* = .66).

Endotoxin measures were obtained from 35 filters. Respirable endotoxin exposure of horses eating hay (3.0 ± 4.2 EU/m^3^) was significantly higher than those eating haylage (0.22 ± 0.11 EU/m^3^; *P* = .003) but not different from horses eating steamed hay (1.19 ± 1.53 EU/m^3^; *P* = .15; Figure 4F).

### 3.5 Effect of forage on clinical score and tracheal mucus

**FIGURE 5:**
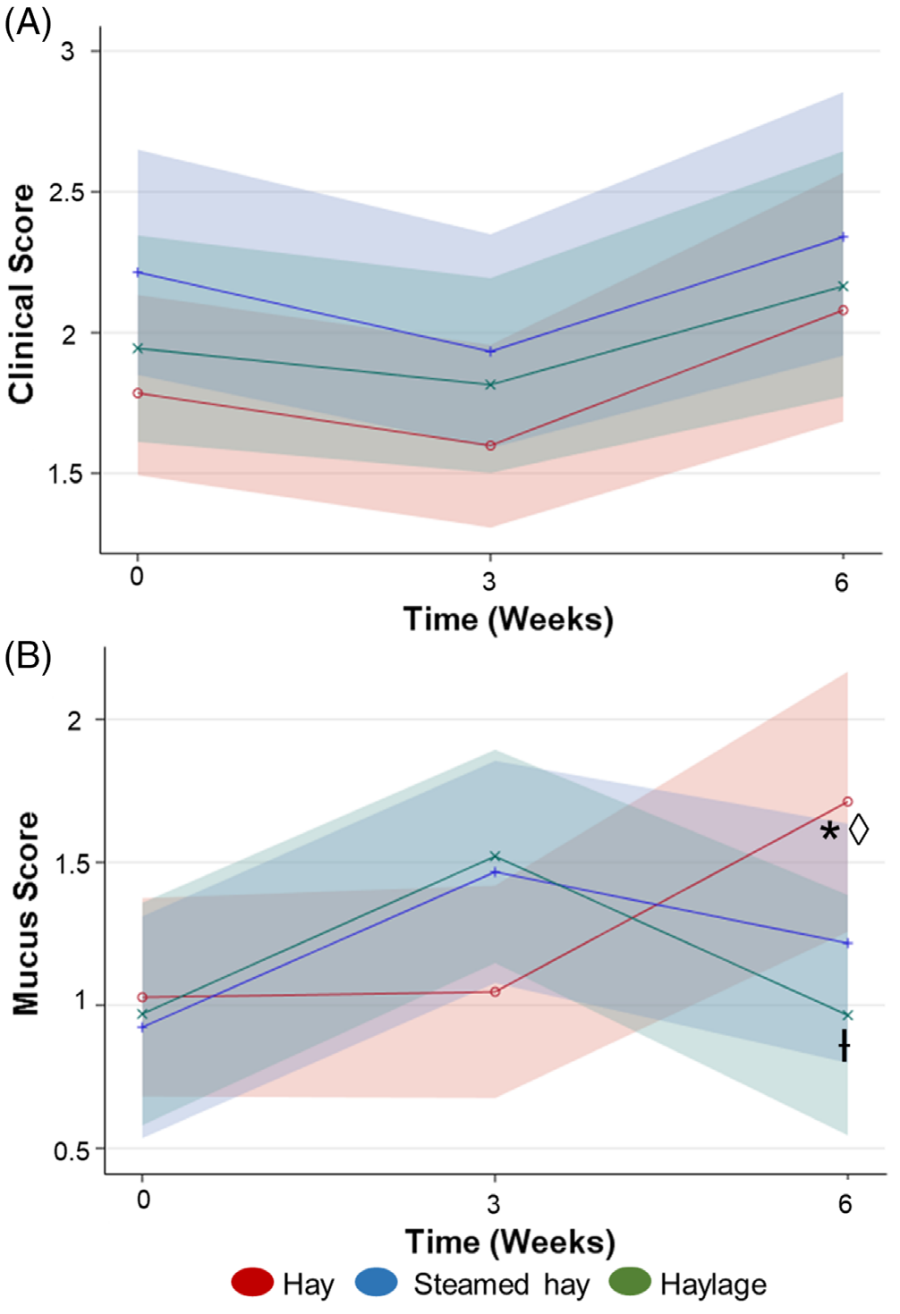
Clinical score and mucus score by forage over time. A) Generalized linear repeated‐measures model of clinical score per forage group over time. B) Generalized linear repeated‐measures model of mucus score per forage group over time. Solid line = mean response, and band = 95% confidence interval. *Different from baseline (*P* = .04). ◊ Different from week 3 (*P* = .04). Ɨ Different from hay at week 6 (*P* = .05). Means, confidence intervals, and P‐values calculated from generalized linear repeated‐measures regression models assuming Poisson distribution and controlling for age and trainer

There was a significant change in clinical score over time regardless of forage assignment (*P* = .03; Figure 5A). Forage assignment had no effect on the clinical score over time (*P* = .98). Respiratory rate was not affected by time (*P* = .60) or forage over time (*P* = .66).

Mucus score was significantly higher in horses eating hay at week‐6 when compared with baseline (*P* = .04) and week‐3 (*P* = .04; Figure 5B). In addition, mucus score was significantly higher at week‐6 in the horses eating hay than those eating haylage (*P* = .05; Figure 5B).

### 3.6 Effect of forage on plasma PUFAs and SPMs

No effect of forage or time was observed on plasma levels of linoleic acid, γ‐linolenic acid, α‐linoleic acid, steridonic acid, eicosadienoic acid, dihome‐γ‐linolenic acid, arachidonic acid, docosadienoic acid, DHA, or EPA (*P* > .1; Table 2). The ratio of EPA to arachidonic acid was significantly higher in horses eating haylage at week‐6 when compared with baseline (*P* = .007), and with horses fed hay and steamed hay at week‐6 (*P* = .002 and *P* = .001, respectively). No effect of time, forage, or forage over time was observed on plasma concentrations of RvD1 (*P* = .59, *P* = .64, and *P* = .99, respectively) or RvE1 (*P* = .24, *P* = .69, and *P* = .37, respectively).

**TABLE 2:** Plasma PUFA proportions by forage over time (data presented as means ± s.e.m.)

PUFA (% of total plasma lipids)	Hay		Steamed hay		Haylage	
	Week 0	Week 6	Week 0	Week 6	Week 0	Week 6
Linoleic acid	42.48 ± 0.61	42.33 ± 0.66	43.39 ± 0.65	42.95 ± 0.66	41.30 ± 0.58	42.55 ± 0.66
γ‐Linolenic acid	0.37 ± 0.03	0.34 ± 0.04	0.30 ± 0.03	0.34 ± 0.04	0.38 ± 0.04	0.33 ± 0.04
⍺‐Linolenic acid	0.49 ± 0.06	0.42 ± 0.06	0.36 ± 0.04	0.29 ± 0.04	0.35 ± 0.05	0.40 ± 0.06
Stearidonic acid	0.32 ± 0.12	Not detected	0.16 ± 0.07	0.24 ± 0.13	0.83 ± 0.57	0.24 ± 0.13
Eicosadienoic acid	0.37 ± 0.06	0.33 ± 0.06	0.44 ± 0.07	0.36 ± 0.06	0.38 ± 0.05	0.55 ± 0.10
Dihome‐γ‐linolenic acid	0.55 ± 0.04	0.54 ± 0.04	0.53 ± 0.04	0.55 ± 0.04	0.57 ± 0.04	0.55 ± 0.04
Arachidonic acid	1.49 ± 0.07	1.44 ± 0.07	1.51 ± 0.07	1.51 ± 0.07	1.52 ± 0.07	1.4 ± 0.07
Docosadienoic acid	0.31 ± 0.16	Not detected	Not detected	0.25 ± 0.18	0.41 ± 0.28	1.02 ± 1.10
DHA	0.70 ± 0.22	0.56 ± 0.15	0.30 ± 0.07	0.42 ± 0.10	0.54 ± 0.14	0.68 ± 0.30
EPA	0.41 ± 0.06	0.38 ± 0.05	0.37 ± 0.05	0.37 ± 0.05	0.49 ± 0.06	0.60 ± 0.13
EPA/arachidonic acid ratio	0.25 ± 0.04	0.25 ± 0.03	0.24 ± 0.04	0.24 ± 0.02	0.34 ± 0.05	0.51 ± 0.07* ^,^ ** ^,^ ***

* Different from baseline (*P* = .007). **Different from horses fed hay at week‐6 (*P* = .002). ***Different from horses fed steamed hay at week‐6 (*P* = .001).

## 4 DISCUSSION

This prospective trial demonstrates that within 3 weeks of dietary modification, racehorses fed haylage have significantly lower BALF neutrophil proportions than horses fed dry hay. After 6 weeks of feeding haylage, horses exhibited a further decrease in BALF neutrophil proportions compared with baseline and horses eating hay. In addition, this trial demonstrates that respirable dust exposure in the breathing zone can be reduced by feeding racehorses steamed hay or haylage in place of dry hay. Finally, plasma EPA to arachidonic acid ratios were higher in horses eating haylage for 6 weeks, while all other PUFAs and SPM concentrations were not different between groups.

At baseline, the forage groups did not differ in overall clinical variables or BALF cytology. Under the criteria from the American College of Veterinary Internal Medicine consensus statement on equine asthma,^3^ 90% of the horses met the cytological criteria for mild asthma at enrollment, similar to the results described in Standardbred racehorses in France (>90%),^5^ and a similar sample of Thoroughbred racehorses in Indiana (80%).^1^ Mast cell inflammation was the most commonly observed BALF cytological profile, comparable to previous results in Thoroughbred racehorses after racing.^1^ In that study, eosinophilic inflammation was observed in only 4% of BALF samples^1^ but in the present study, eosinophilic inflammation was observed in 19% of baseline measurements. This difference could be explained by the higher number of 2‐year‐old horses sampled in the present study, since eosinophilic inflammation appears to be more frequent in younger horses.^2^

None of the horses that withdrew from the study evidenced any adverse effect from either the feeding or sampling protocol. Haylage and steamed hay were well accepted, and no adverse effects or changes in fecal consistency were observed during the study. Botulism is associated with feeding poorly preserved haylage to horses.^30^ We advised horsemen to discard haylage with visible mold growth, and haylage bales were fed within 3 days of opening.^31^, ^32^ In addition, vaccination against *Clostridium botulinum* type B was offered free of charge. The hygienic quality of both hay and haylage can vary widely depending on composition and conditions during production,^33^, ^34^, ^35^ and there are relatively few published data comparing measures of dust exposure of horses while eating different forages.

In the current study, respirable dust exposures in the breathing zone were lower in horses eating steamed hay or haylage when compared with those eating hay, with exposure reduced by approximately 30%. The mean respirable dust exposure measured in the breathing zone of horses eating hay (0.08 mg/m^3^) was comparable with Thoroughbred racehorses eating hay and bedded on sawdust (0.06 ± 0.09 mg/m^3^),^1^, ^2^ and a pony fed hay and bedded on shavings (0.06 ± 0.04 mg/m^3^).^36^ Exposures when the pony was fed haylage (0.03 ± 0.01 mg/m^3^) were lower than those measured in the current study (0.05 ± 0.02 mg/m^3^); however, breathing‐zone measures of exposure vary markedly between horses,^2^ and interpretation of measurements taken from a single animal should be limited. Exposure of horses eating steamed hay on pasture (0.0015 mg/m^3^)^37^ was lower than that observed in this study (0.06 ± 0.02 mg/m^3^), a difference that might be explained by better air quality outdoors compared with stalls. Additionally, differences in the LOD between the studies might be important, especially considering the high number of measurements that were under the LOD in horses eating haylage (78%) and steamed hay (68%) in the current study. An extended sampling time of 6 hours would achieve a lower LOD.

Average PM_10_ and PM_10‐2.5_ exposures were lower for the horses eating haylage when compared with steamed hay and hay. Also, PM_10_ was significantly lower in horses eating steamed hay when compared with horses eating hay. In racehorses, higher exposure to breathing zone PM_10_ is associated with increased tracheal mucus accumulation and higher neutrophil proportions in tracheal wash.^38^ In humans, increased PM_2.5_ and PM_10_ exposures are associated with exacerbation of asthma.^39^ In addition, PM_10‐2.5_ exposure is related to the development of asthma in children,^40^ such that for each 0.001 mg/m^3^ increase in the average coarse particle exposure, the prevalence of asthma increases by 0.6%. The current study demonstrates that substituting a low dust forage such as steamed hay or haylage in place of hay might reduce exposure to PM_10_ by an average of 44% and 66%, respectively.

Respirable β‐glucan exposure did not vary with forage type in the current study. In a previous study of Thoroughbred racehorses that used the same methodology, the mean β‐glucan exposure (55.5 ± 66.2 pg/m^3^)^1^ was similar to the exposure measured in horses fed hay in the present study (48.3 ± 4.2 pg/m^3^).

Respirable endotoxin concentrations were significantly lower in horses eating haylage when compared with horses eating hay. Airborne endotoxin exposure for a single horse eating haylage is similar to horses on pasture, and significantly lower than horses fed hay.^41^ Eating steamed hay yields a 15‐fold lower respirable endotoxin activity when compared with eating hay.^37^ In the current study, no difference in respirable endotoxin concentration was observed between horses fed hay or steamed hay. The median endotoxin exposure of horses fed hay was 1.2 EU/m^3^ in the current study, while median exposures reported for horses eating hay range from 2.2 to 7080 EU/m^3^.^1^ The variability between endotoxin exposures in these studies can be explained by the large effects that methods of sampling and handling can have on endotoxin measurements^42^ as well as the environmental factors during hay harvest.^34^

Horses fed haylage exhibited a transient decrease in BALF mast cell proportions. Mast cell inflammation has been positively associated with respirable β‐glucan.^1^ However, β‐glucan measurements were below the assay detection limit in many of the samples in the current study. A more sensitive assay of respirable β‐glucan might have helped to explain the observed changes in mast cell proportions. In horses eating steamed hay, a significant decrease was observed in BALF eosinophil proportions at week‐3 when compared with baseline and horses eating hay. This change was transient and was no longer significant at week 6. The clinical relevance of this change in eosinophil proportions is unclear because mean BALF eosinophil proportions were under the 1% threshold for diagnosis of eosinophilic inflammation in each group.^3^

Clinical score changed over time independent of forage, but the changes were small, <0.5 points on a 21‐point scale, and not considered clinically relevant. Furthermore, the clinical score at baseline was not different from the score at week‐6.

Endoscopic mucus score increased over time when horses were eating hay, and the mucus score of horses fed haylage at week‐6 was lower than horses fed hay. While mucus scores are affected by the time elapsed between exercise and endoscopy,^43^ this timing was not standardized in the present study. However, these results are consistent with a median mucus score of 2 that was reported in a study of Thoroughbreds examined ~1 hour after racing where all horses were fed dry hay.^1^

Lower BALF neutrophil proportions at week‐3 and week‐6 were observed in horses fed haylage compared with those fed hay. This study documents the beneficial effects of low‐dust forage feeding on BALF cytology in racehorses with naturally occurring airway inflammation. Horses fed haylage has a rapid reduction in BALF neutrophil proportions, while those fed hay pellets do not, despite equivalent PM exposures.^44^ When fed haylage, horses with severe asthma can maintain clinical remission and low BAL neutrophil proportions (<10%) for months.^45^ A similar beneficial effect was observed in horses with mild asthma in the current study. Considering that small changes in BALF neutrophil proportion can affect racing performance,^1^ the decrease in BALF neutrophils observed in horses fed haylage is likely to be clinically relevant for racehorses.

In the United States, racehorses are frequently fed hay as their sole forage with no access to pasture. Dry hay has lower Ω‐3 content when compared with haylage and fresh grass.^22^ Ω‐3 are the main precursors for the production of SPM,^19^ molecules that are crucial in the resolution of inflammation.^46^ Furthermore, EPA and arachidonic acid compete for the same enzymatic pathways.^47^ The results of this competition determine whether SPM or inflammatory mediators are produced. Horses eating haylage had significantly increased plasma EPA to arachidonic acid ratios compared with horses eating steamed hay or dry hay; however, plasma levels of SPM such as RvD1 and RvE1 did not vary with forage or time. Other, unmeasured SPM might have been involved in the enhanced resolution of airway inflammation when horses were fed haylage.^48^

In horses with severe asthma, the addition of an Ω‐3 supplements to a low dust diet achieves faster improvement in clinical signs and BALF cytology compared with horses fed a low‐dust diet with placebo.^12^ Furthermore, plasma DHA concentrations are significantly higher in the horses fed algae‐derived Ω‐3 supplement. In humans, diets with higher content of DHA and EPA might be protective against inflammatory diseases like asthma while other studies do not find this association.^49^ EPA competes with arachidonic acid as a substrate for enzymes and is converted to SPM,^50^ and in vitro studies of alveolar macrophages from humans with asthma reveal that EPA is a more potent inhibitor of pro‐inflammatory mediators than DHA.^51^ Furthermore, EPA is an important precursor of the resolvin E‐series of SPM,^46^ and the differences in plasma EPA to arachidonic acid ratios between horses eating haylage and those eating dry or steamed hay might explain the differences in BALF neutrophil proportions observed in this study. Human athletes supplemented with Ω‐3 (3.2 g EPA and 2.2 g DHA) have a suppression of exercise‐induced bronchoconstriction associated with a decrease in circulating inflammatory cytokines in vitro and production of leukotrienes from neutrophils in vitro.^52^

A limitation of our study was that most of the horses in the study were provided by a single trainer. Compliance with the study design required cooperation and extra work hours from staff that some trainers found burdensome, which limited their participation. In addition, it was impossible to mask investigators to forage assignment. While this information could potentially bias investigators, the measures of exposure, PUFA, and SPM are objective. BALF differential cell counts might be more vulnerable, as bias can be introduced in selection of cells to enumerate. However, counting at least 600 cells in a systematic manner for each BALF was performed to minimize the risk of such bias.

## CONFLICT OF INTEREST DECLARATION

Authors declare no conflict of interest.

## OFF‐LABEL ANTIMICROBIAL DECLARATION

Authors declare no off‐label use of antimicrobials.

## INSTITUTIONAL ANIMAL CARE AND USE COMMITTEE (IACUC) OR OTHER APPROVAL DECLARATION

The Purdue University Animal Care and Use Committee (protocol #1111000181) and the Indiana Horse Racing Commission approved all procedures.

## HUMAN ETHICS APPROVAL DECLARATION

Authors declare human ethics approval was not needed for this study.

## Supporting information


**Table S1:** Distribution and link functions used in data analysis for each outcome measure.

**Table S2:** Description of housing type and forage assignment for each of the 43 horses throughout the trial.
