# Supplementary material for: Effects of low‐dust forages on dust exposure, airway cytology, and plasma omega‐3 concentrations in Thoroughbred racehorses: A randomized clinical trial
Source: J Vet Intern Med. 2022 Dec 7;37(1):338–48. doi: 10.1111/jvim.16598 (PMC9889630; doi:10.1111/jvim.16598)
Supplement: Supplementary file 1 — Table S1: Distribution and link functions used in data analysis for each outcome measure. [file JVIM-37-338-s001.pdf]

Outcome variable	Distribution	Link function
Exposure variables	Lognormal	Identity
Clinical score	Poisson	Log
Tracheal mucus score	Poisson	Log
Respiratory rate (breaths per min)	Poisson	Log
Inflammatory cell proportions	Binomial (trials/events)	Logit
Polyunsaturated fatty acids	Lognormal	Identity
Specialized pro-resolving mediators	Lognormal	Identity
