# Supplementary material for: Effects of low‐dust forages on dust exposure, airway cytology, and plasma omega‐3 concentrations in Thoroughbred racehorses: A randomized clinical trial
Source: J Vet Intern Med. 2022 Dec 7;37(1):338–48. doi: 10.1111/jvim.16598 (PMC9889630; doi:10.1111/jvim.16598)
Supplement: Supplementary file 2 — Table S2: Description of housing type and forage assignment for each of the 43 horses throughout the trial. [file JVIM-37-338-s002.pdf]

Number	Trainer	Barn type	Sex	Forage	Study Protocol Completed
1	1	Enclose	Stallion	Hay	Yes
2	1	Enclose	Stallion	Hay	No
3	2	Shed Row	Gelding	Hay	Yes
				Steamed Hay	Yes
				Haylage	Yes
4	2	Shed Row	Mare	Haylage	No
5	2	Shed Row	Gelding	Steamed Hay	Yes
				Haylage	No
6	2	Shed Row	Mare	Steamed Hay	Yes
				Haylage	Yes
				Hay	No
7	2	Shed Row	Stallion	Hay	No
8	2	Shed Row	Mare	Steamed Hay	Yes
				Haylage	Yes
				Steamed Hay	No
9	2	Shed Row	2	Hay	Yes
				Steamed Hay	W3
10	2	Shed Row	1	Hay	Yes
				Steamed Hay	Yes
				Haylage	No
11	2	Shed Row	2	Steamed Hay	Yes
				Haylage	Yes
12	2	Shed Row	1	Steamed Hay	Yes
				Haylage	Yes
				Hay	Yes
13	2	Shed Row	2	Hay	No
14	2	Shed Row	2	Haylage	Yes
15	2	Shed Row	2	Hay	No
16	2	Shed Row	2	Steamed Hay	Yes
				Hay	Yes
				Haylage	Yes
17	1	Enclose	1	Hay	No
18	1	Enclose	Stallion	Hay	No
19	1	Enclose	1	Hay	No
20	1	Enclose	1	Hay	No
21	2	Shed Row	Stallion	Haylage	Yes
22	2	Shed Row	2	Steamed Hay	Yes
				Haylage	Yes
23	2	Shed Row	2	Steamed Hay	Yes
				Haylage	No

				Hay	Yes
				Haylage	Yes
24	2	Shed Row	1	Steamed Hay	No
				Haylage	No
				Hay	Yes
				Haylage	Yes
25	2	Shed Row	1	Hay	Yes
				Steamed Hay	Yes
26	2	Shed Row	2	Haylage	No
27	3	Free span central aisle	2	Steamed Hay	Yes
28	3	Free span central aisle	1	Steamed Hay	Yes
29	4	Shed Row	2	Haylage	Yes
30	4	Shed Row	2	Haylage	Yes
31	2	Shed Row	1	Hay	Yes
				Steamed Hay	Yes
32	2	Shed Row	1	Hay	Yes
				Steamed Hay	Yes
				Haylage	Yes
33	2	Shed Row	1	Hay	Yes
34	2	Shed Row	1	Steamed Hay	Yes
				Haylage	Yes
35	2	Shed Row	1	Steamed Hay	No
36	2	Shed Row	2	Steamed Hay	Yes
				Haylage	Yes
37	2	Shed Row	2	Hay	Yes
				Haylage	Yes
38	2	Shed Row	1	Hay	No
39	2	Shed Row	2	Hay	Yes
				Haylage	Yes
40	2	Shed Row	1	Hay	No
41	2	Shed Row	1	Hay	No
42	2	Shed Row	1	Hay	Yes
43	2	Shed Row	1	Hay	Yes
